# Dried Root of *Rehmannia glutinosa* Prevents Bone Loss in Ovariectomized Rats

**DOI:** 10.3390/molecules18055804

**Published:** 2013-05-17

**Authors:** Dong Wook Lim, Yun Tai Kim

**Affiliations:** Functionality Evaluation Research Group, Division of Metabolism and Functionality Research, Korea Food Research Institute, Seongnam 463-746, Korea

**Keywords:** dried root of *Rehmannia glutinosa*, osteoporosis, ovariectomized rat model

## Abstract

Dried root of *Rehmannia glutinosa* is a kidney-tonifying herbal medicine with a long history of safe use in traditional folk medicine for the treatment of joint diseases. This study was conducted to investigate prevention of bone loss by a standardized dried root of *R. glutinosa* in an ovariectomized (OVX) rat model of osteoporosis. The OVX groups were divided into five groups treated with distilled water, 17*β*-estradiol (E2 10 µg/kg, once daily, i.p) and dried root of *R. glutinosa* extracts (DRGE 30, 100, and 300 mg/kg, twice daily, p.o) for eight weeks. We measured the body, organs, and uterus weights, and femur and lumbar vertebrae bone mineral density (BMD), serum alkaline phosphatase (ALP), estradiol levels. The treatments with DRGE 300 mg/kg significantly inhibited BMD decrease in the femur and lumbar (17.5% and 16.4%, *p* < 0.05, respectively) by OVX without affecting the body, organs, and uterus weights. Also, serum ALP level in the DRGE 300 mg/kg treated group was significantly decreased, but the estradiol level did not change in serum of the DRGE 300 mg/kg treated group. These results show that DRGE is able to prevent OVX-induced bone loss without influencing hormones such as estrogen.

## 1. Introduction

Osteoporosis is characterized by a reduction in bone mass and microarchitectural deterioration of bone tissue, resulting in skeletal fragility and susceptibility to fractures [1]. The most common type of osteoporosis is the bone loss associated with estrogen deficiency at postmenopausal women [2]. In addition, secondary hyperparathyroidism, associated with calcium or vitamin D deficiency, may accelerate bone loss and increase the risk of developing osteoporosis. Both estrogen deficiency and secondary hyperparathyroidism are associated with a primary increase in bone resorption and an impaired bone formation response [3].

Hormone replacement therapy (HRT) has proven to be efficacious in preventing bone loss and reducing the incidence of skeletal fractures in postmenopausal women [4]. However, long-term HRT increases the high risk of breast and endometrial cancer, thromboembolic events and vaginal bleeding [5]. Concerns about the adverse side effects of HRT have led to interest in the anti-osteoporotic activity of natural products.

*Rehmannia glutinosa* Libosch, which belongs to the family of Scrophulariaceae, is one of the earliest known and most important edible crude herbs used for various medicinal purposes in East Asia. There are two types of *R. glutinosa* used as medicinal herbs, named Gun-Ji-Whang (non-processed root; dried rehmannia root), and Sook-Ji-Whang (processed root; steamed rehmannia root) in Korean according to the processing method [6]. Dried or steamed root of *R. glutinosa* have been used to reduce fever, activate blood circulation, tonify the kidney, and for *Yin* deficiency syndrome, and they are used in quite different therapeutic applications and the choice is strictly defined in Traditional Chinese Medicine (TCM) [7]. The root of *R. glutinosa* has also been reported to possess anti-tumor [8], anti-stress [9], anti-thrombic [10], and hypo-glycemic [11] effects. The major active components of the root of *R. glutinosa* are iridoid compounds such as catalpol and dihydrocatalpol, while other components are phenol glycoside ionones, flavonoids, amino acids, inorganic ions, microelements, which are responsible for its diverse bioactivities [7].

It was reported that steamed root of *R. glutinosa* stimulates the proliferation of osteoblasts, while inhibiting the generation and resorptive activities of osteoclasts in bone metabolism [12]. The herbal formulationYukmi-jihang-tang, consisting of seven kidney-nourishing herbs was reported to reduce bone resorption both in *in vitro* and *in vivo* by inhibition of phosphorylation of peptide substrates [13,14]. Recently, catalpol from fresh root of *R. glutinosa* has been reported to promote the proliferation of osteoblasts of MC3T3-E1 cells.

Although dried and steamed root of *R. glutinosa* are used in quite different therapeutic applications in TCM, dried root of *R. glutinosa* also might have potential effects in regulating bone metabolism because both of dried and steamed root of *R. glutinosa* have related main active constituents [15]. However, dried root of *R. glutinosa* has not received much attention concerning bone metabolism. Prevention of bone loss by dried root of *R. glutinos*a in an ovariectomized (OVX) rat model has not been investigated yet. We have studied the acute effects of dried and steamed root of *R. glutinosa* (50% EtOH extraction) in a OVX rat model (unpublished data). Our findings demonstrated that four weeks of treatment with dried root of *R. glutinosa* extracts (DRGE) significantly decreased the BMD loss in femur compared to the control group and that the BMD loss was not significantly decreased in animals given steamed root of *R. glutinosa* extracts (SRGE), however, this could have simply been because the changes in BMD in the DRGE treated group were less variable than in the SRGE treated group. That said, DRGE was efficacious than an equivalent dose of SRGE in the OVX rat model, so we did not use SRGE to perform the long-term experiments, and have now focused on whether long-term DRGE treatment decreases bone loss in OVX rats. Thereby, we have performed the DRGE treatments in rats in pre-osteoporosis state.

In the present study we examined the prevention of bone loss of a standardized dried root of *R. glutinosa* in an OVX rat model. Body weight and bone mineral density (BMD) of femur and lumbar vertebrae were determined weekly using dual energy X-ray absorptiometry (DXA). Serum alkaline phosphatase (ALP) concentration was measured by a biochemistry analyzer. Serum estradiol levels were also determined by a radioimmunoassay (RIA) kit.

## 2. Results and Discussion

### 2.1. HPLC Chromatograms for Standardization of DRGE

**Figure 1:**
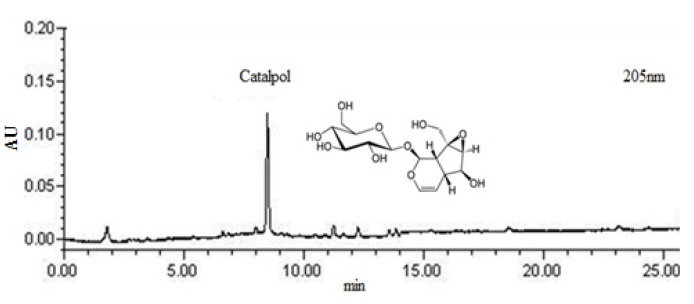
2-D HPLC chromatograms for standardization of DRGE.

Dried root of *R. glutinosa* extracts (DRGE) was monitored at 205 nm for catalpol (Figure 1). The content of catalpol was calculated for standardization. DRGE was standardized to contain 5.4 mg/g catalpol.

### 2.2. Bone Mineral Density of the Femur and Lumbar Vertebrae in Treatments of DRGE

**Figure 2:**
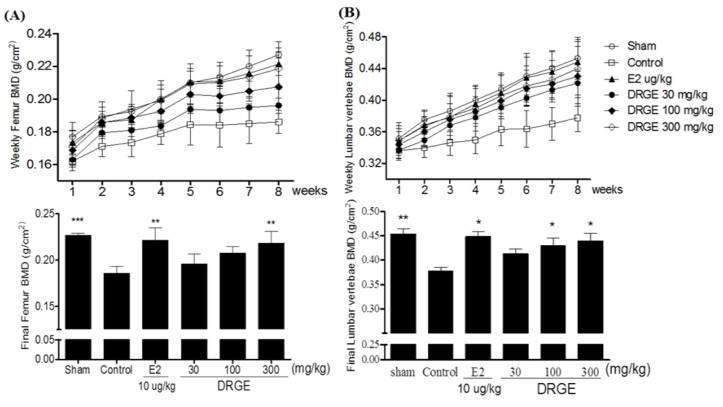
(**A**) Effects of DRGE on BMD in right femur and (**B**) lumbar vertebrae (g/cm^2^) of OVX rats by dual energy X-ray absorptiometry (DXA). These BMD values were determined weekly during the experimental period. Data are mean ± SD values (n = 12 per group). * *p* < 0.05, ** *p* < 0.01, significantly difference from the OVX-control group.

Three weeks after the OVX operation, OVX groups showed a significant decrease in the right femur bone mineral density (BMD) and lumbar vertebrae (1–4 regions) compared to the sham group (*p* < 0.05). After eight weeks of treatments, the final femur BMD of the 300 mg/kg DRGE-treated groups were significantly higher than that of the OVX-control group (17.5%, *p* < 0.01 *vs*. control, Figure 2A). Also, the lumbar vertebrae BMD of the DRGE 100 and 300 mg/kg-treated groups were significantly higher compared to the OVX-control group (14% and 16.4%, respectively, *p* < 0.05 *vs*. control, Figure 2B).

### 2.3. Weekly Body Weight in DRGE Treatments

**Figure 3:**
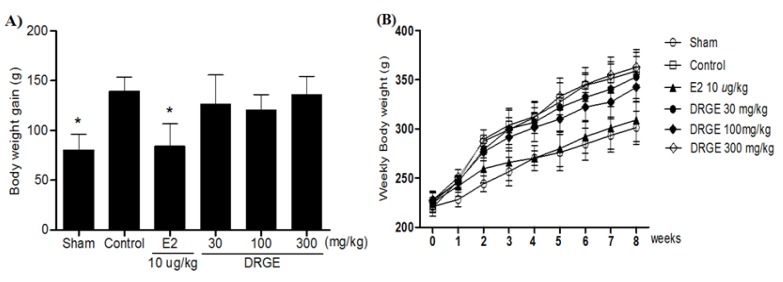
(**A**) Effects of DRGE on body weight gain and (**B**) body weight (g) in OVX rats. The body weight was recorded weekly during the experimental period. The body weight gain was calculated by the equation: final body weight - initial body weight. Data are mean ± SD values (n = 12 per group). * *p* < 0.05, significantly difference from the OVX-control group.

Body weights increased over time in all groups, but body weights increased significantly more in the OVX groups alone than in sham groups. A significant difference in body weight was observed between the E2 10 µg/kg treated group and the OVX-control group by two weeks after initiating administration. The body weight gain of the E2 10 µg/kg treated group was also significantly less than that of the OVX-control group. However, there was no significant difference in the body weight and body weight gain of DRGE-treated groups during the experimental period (Figure 3).

### 2.4. Uterus and Organ Index in Treatments of DRGE

**Figure 4:**
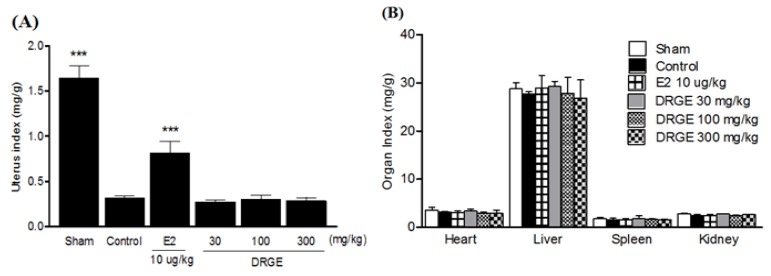
(**A**) Effects of DRGE on change in uterus and (**B**) organ index (mg/g). Uterus and organs were dissected, washed with saline, and immediately weight for analysis. Data are mean ± SD values (n = 12 per group). * *p* < 0.05, significantly difference from the OVX-control group.

OVX caused atrophy of uterine tissue, indicating the success of the surgical procedure and in the E2 10 µg/kg treated group the uterus index (mg/g) increased significantly compared to the OVX-control group. However, DRGE-treated groups did not show an effect on the uterus index following OVX (Figure 4A). The index of heart, liver, spleen, and kidney was not significantly different in each group either (Figure 4B).

### 2.5. Serum ALP and Estradiol Concentration in Treatments of DRGE

**Figure 5:**
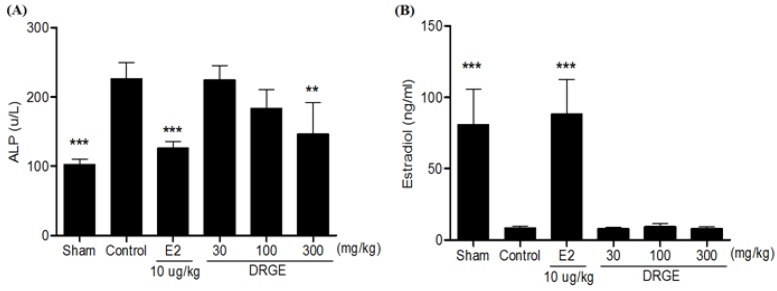
(**A**) Effects of DRGE on ALP and (**B**) estradiol concentrations in OVX rat. At the end of the treatment period, blood sample was collected via abdominal aorta. Serum ALP and estradiol were measured by biochemical analyzer. Data are mean ± SD values (n = 12 per group). ** *p* < 0.01, ** *p* < 0.001, significantly difference from the OVX-control group.

ALP level in the OVX-control group was significantly higher compared to the sham group. After eight treatments, the DRGE (300 mg/kg) treated group displayed significantly lower serum ALP levels compared to the OVX-control group (Figure 5A). In addition, the DRGE 300-treated group was not significantly different from the OVX-control group (Figure 5B).

### 2.6. Discussion

Our findings demonstrate that eight weeks of treatment with DRGE significantly decreased the BMD loss in the femur and lumbar vertebrae and inhibited the increase in serum ALP levels compared to the OVX control group without the influence of hormones such as estrogen.

Bone loss caused by estrogen deficiency in both humans and experimental animals is primarily due to an increase in osteoclastic bone resorption [16]. OVX rats, which exhibit most of the characteristics of human postmenopausal osteoporosis [17] have been widely used as a model for the evaluation of potential osteoporosis treatments [18].

Like previous reports, our OVX resulted in significantly decrease in the femur and lumbar vertebrae BMD after eight weeks. This BMD loss was accompanied by a significant increase in bone remodeling, as was evidenced by the enhanced bone turnover makers. An increase in ALP serum levels, the most widely used biochemical bone turnover marker [19], was observed in OVX rats [20]. Although we did not determine the 3-D architecture of trabecular bone within the distal metaphyseal femur region, oral administration of DRGE at dosages of 300 mg/kg significantly decreased the BMD loss in the femur and lumbar vertebrae, which was reflected by the decrease in ALP serum levels compared to the OVX-control group. These results suggest that DRGE decreases bone loss by inhibiting bone remodeling in OVX rats.

OVX dramatically increases body weights, while E2 treatment prevents normal levels completely [21]. Although the mechanisms by which OVX induces an increase in body weight are not clear, estrogen deficiency induced body fat accumulation and subsequently caused an increase in body weight [22]. Heine *et al*. demonstrated that estrogen receptor (ER) knockout mice have higher fat mass and lower energy expenditure than wild-type mice [23]. Estrogen may be involved directly in rat energy metabolism by binding to ER within the abdominal and subcutaneous fat tissues [24]. In our results, DGRE did not affect OVX-induced body weight gain, and serum estradiol concentration. These results suggest that DGRE did not display estrogen-like activity in OVX rats.

Estrogen expresses its activities by binding to different ERs, ERα and ERβ. ERβ is more abundant than ERα in bone tissue while ERα is mainly distributed in reproductive cells and is the dominant receptor mediating the most obvious effects of E2 in breast and uterus [25]. As mentioned above, DRGE decreased bone loss without resulting in an increased uterus weight. Although measurement of uterus weight was relatively crude, these results indicate that DRGE might show a higher affinity for ERβ than for ERα that produces optimal action in preventing bone loss without stimulating an unwanted proliferation of the uterine tissues. Consistent with our finding from the E2 treated group, the DRGE might have anti-osteoporotic effects in OVX rats, without the influence of hormones such as estrogen. However, further mechanistic studies are needed to clarify whether the prevention of bone loss effects of DRGE may be elicited by regulating the expressions of ERβ.

There have been studies on the biological activities of iridoid glycosides, which are potent antioxidants and free radical scavengers [26]. It has been demonstrated that oxidation-derived free radicals increase bone resorption by promoting osteoclastic differentiation [27]. Catalpol has also demonstrated promotion of proliferation and differentiation of MC3T3-E1 cells, a mouse osteoblast cell line, *in vitro*. The effects of DRGE on bone thus appear to be related to its high contents of the iridoid glycosides such as catalpol.

Kim *et al*. demonstrated that that *R. glutinosa* inhibits the secretion of both interleukin-1 (IL-1) and tumor necrosis factor-a (TNF-α) from mouse astrocytes [28]. These cytokines are well known regulators of bone metabolism. IL-1 is known as a highly potent bone resorptive cytokine [29], and TNF-α appears to synergize with IL-1h in their ability to increase bone resorption [30]. From the above reports, it is also hypothesized that *R. glutinosa* might have potential effects in regulating bone metabolism.

## 3. Experimental

### 3.1. Sample Preparation and HPLC Analysis

Dried root of *R. glutinosa* was purchased Yaksudang Co. (Seoul, Korea).The sample was identified by Dr. HeeSoon Shin and a voucher specimen (#NP-1031) was deposited in the Functionality Evaluation Research Group, Korea Food Research Institute, Seongnam, Korea. The dried root of *R. glutinosa* (300 g) was extracted with 50% ethanol (3,000 mL) for 3 h at 80 °C in a reflux apparatus. The extracts were filtered and concentrated under reduced pressure, and samples were lyophilized to yield a dark yellow powder. The yield of dried root of *R. glutinosa* extract (DRGE) was 13.8%. The quantitative authentication of DRGE was performed by a high performance liquid chromatography (HPLC) system equipped with a Waters 1525 pump, a 2707 auto sampler and a 2998 PDA detector. The chromatic separation was achieved at 30 °C on Waters Sunfire™ C18 (250 mm × 4 mm i.d., 5 μm particle size) column. DRGE was monitored at 205 nm for catalpol. The run time was set at 30 min and the flow rate was 1.0 mL/min and the sample injection volume was 10 μL. Mobile phases A and B were acetonitrile and water, respectively. Gradient elution was as follows: 0–10 min 0–10% solvent A, 10–20 min 15–45% solvent A, 20–30 min 45% solvent A. The content of catalpol was calculated for standardization. DRGR was standardized to contain 5.4 mg/g catalpol.

### 3.2. Animals and Treatments

Female Sprague-Dawley (SD) rats, 8-weeks old, were purchased from Samtako, Gyeonggi-do, Korea. Animals were housed at two rats per cage in an air-conditioned room at 23 ± 1 °C, 55–60% relative humidity, and a 12 h light/dark cycle (07:00 lights on, 19:00 lights off), and were given a laboratory regular rodent diet. All animal experiments were carried out according to the guidelines of the Korea Food Research Institutional Animal Care and Use Committee (KFR-M-13003). After acclimatization for 1 week, 9-week-old female SD rats were anesthetized with 2% of isoflurane and ovaries were removed bilaterally. A sham operation, during which the ovaries were just touched with forceps, was performed on the sham group. A recovery period of 1 week after surgery, rats were divided into five following treatment groups: (1) sham + vehicle, (2) OVX + vehicle, (3) OVX + 17*β*-estradiol (E2, 10 μg/kg once daily, i.p), (4) OVX + DRGE 30 mg/kg, (5) OVX + DRGE 100 mg/kg, (6) OVX + DRGE 300 mg/kg. DRGE at a dosage of 30 mg/kg in rats corresponds to 1.8 g DRGE/60 kg-weighed human subject, where DGRE extracted from approximately 13 g of the DRGE raw material. Finally, we decided the dosages of DRGE, *i.e.*, 30, 100, and 300 mg/kg, separated by three time intervals. DRGE was dissolved in distilled water for oral administration at the desired doses in a volume of 5 mL/kg twice daily at 08:00 am and 08:00 pm. E2 dissolved in distilled water, with 1% dimethyl sulfoxide (DMSO) and 0.1% Tween 20. All groups were treated for eight weeks. During the experimental period, body weight and femur and lumbar vertebrae bone mineral density (BMD) were determined weekly. At the end of the treatment period, the rats were fasted for 12 h, and blood was collected via the abdominal aorta. Uterus tissue and other organs were dissected, washed with saline solution, and weighted for analysis. Uterus and organ indexes (mg/g) were calculated by dividing the uterus and organ weights by the body weight.

### 3.3. Bone Mineral Density Measurements

The BMD of femur was measured by a PIXImus (GE Lunar PIXImus, GE Healthcare, WI, USA), dual energy X-ray absorptiometer (DXA), equipped with appropriate software for bone density assessment in small laboratory animals. Calibration of the instrument was conducted as recommended by the manufacturer. Quality control with BMD (0.0553 g/cm^2^) and percentage fat composition (16.7%) of the phantom were also performed each time the instrument was switched on. All rats were placed in the same direction.

### 3.4. Serum ALP and Estradiol Analysis

The serum samples were prepared by centrifugation of the collected blood samples (1,013 *g* for 15 min at 4 °C), then stored at −80 °C for biochemical determinations. Serum ALP concentrations were measured by VetTest 8008 (IDEXX Lab Inc., Westbrook, ME, USA). Serum hormone level was determined by radioimmunoassay (RIA). The estradiol RIA was performed according to the instructions accompanying a Coat-a-Count kit (Diagnostic Products, Los Angeles, CA, USA).

### 3.5. Statistical Analysis

All data were presented as the mean ± standard deviation (SD). The effects of different treatments were compared by one-way ANOVA test, followed by the post-hoc Tukeytest for multiple comparisons using GraphPad Prism 5 (GraphPad Software Inc., La Jolla, CA, USA). *p* < 0.05 was considered statistically significant.

## 4. Conclusions

In conclusion, DRGE is able to prevent OVX-induced in bone loss without the influence of hormones such as estrogen, suggesting that DRGE may be a reasonable natural alternative for the prevention of postmenopausal osteoporosis. However, further detailed mechanistic investigation of the anti-osteoporotic-effects of DRGE on bone metabolism is required.
